# The *ebony* Gene in Silkworm Black Pupae Significantly Affects 30 K Proteins During the Pupal Stage

**DOI:** 10.3390/genes15121560

**Published:** 2024-11-29

**Authors:** Runhuan Yang, Huiduo Guo, Juan Sun, Tao Gui, Xinyu Li, Heying Qian, Anli Chen

**Affiliations:** 1 Jiangsu Key Laboratory of Sericultural and Animal Biotechnology, School of Biotechnology, Jiangsu University of Science and Technology, Zhenjiang 212100, China; yrh0905@163.com (R.Y.); guohuiduo1991@126.com (H.G.); sj15062884263@163.com (J.S.); 15250895410@163.com (T.G.); 2 Key Sericultural Laboratory of Shaanxi, Ankang University, Ankang 725000, China; lxylzc2004@163.com; 3 Key Laboratory of Silkworm and Mulberry Genetic Improvement, Ministry of Agriculture and Rural Affairs, Sericultural Scientific Research Center, Chinese Academy of Agricultural Sciences, Zhenjiang 212100, China

**Keywords:** *ebony*, 30 KPs, NisBP, CRISPR/Cas9, *Bombyx mori*

## Abstract

**Background/Objectives**: The body color and patterns of insects play important roles in foraging, evading predators, mating, thermoregulation, and environmental adaptation. During the rearing of the QiufengN silkworm strain, a mutant with black pupal cuticle (QiufengNBP) was discovered. Preliminary map-based cloning and sequence analysis indicated that the *ebony* gene might significantly influence the formation of the black pupa mutant and the expression of 30K proteins. This study aims to determine the function of the *ebony* gene and its effect on the expression of the 30K protein during the pupal stage; **Methods and Results**: We employed CRISPR/Cas9 gene-editing technology to knock out the *ebony* gene in the Nistari strain, resulting in individuals with black pupae, named Nistari Black Pupa (NisBP). This confirmed that the *ebony* gene plays a crucial role in black pupa formation. Two-dimensional electrophoresis (2-DE) analysis of the pupal cuticle of NisBP and its wild-type Nistari found that the *ebony* gene has a significant impact on the expression of 30K proteins, which are vital for embryonic development and serve as key storage proteins; **Conclusions**: This study is the first to demonstrate that the *ebony* gene affects the expression of 30K proteins, laying the foundation for further research on their functions and providing insights into the developmental mechanisms of silkworms.

## 1. Introduction

Melanin is produced by melanocytes in insects through the melanin metabolic pathway, and abnormal metabolism of this pathway is the primary reason for the occurrence of melanization in insects. The melanin synthesis metabolic pathway is as follows: Using tyrosine as a substrate, it is converted into dihydroxyphenylalanine (DOPA) under the action of tyrosine hydroxylase (TH). DOPA is then converted into DA by dopamine decarboxylase (DDC) [1]. The *ebony* gene encodes an NBAD synthase that modifies DA to NBAD, controlling the formation of black melanin and brown melanin through phenol oxidase and cofactor systems during the development of the cuticle [2,3,4,5]. The linkage mapping of insect melanin synthesis genes shows that the *yellow* and *ebony* genes are located near the *ch* and *so* loci, respectively. In the color patterns of lepidopteran insects, the *yellow* gene promotes melanization, while the *ebony* gene inhibits it; both are crucial in the melanin synthesis pathway [6].

Silkworm (*Bombyx mori*) is an economically important insect and Lepidopteran model organism with over 20 known types of melanization mutations occurring at various stages of its metamorphosis [7]. One such melanization mutation, the *mln* mutant, exhibits black pigmentation in the hardened areas of the head, thoracic legs, and anal plates during the larval stage and throughout the pupal and adult stages. This type of mutation, which shows blackening across all developmental stages, is rare in silkworms. Research has shown that it results from a mutation in the *Bm-iAANAT* gene located on chromosome 18, which disrupts the acetyltransferase domain, causing an excessive accumulation of dopamine (DA) and resulting in the melanization phenotype of the *mln* mutant [8,9]. The *bp* mutation is another silkworm pupal blackening mutation that only exhibits the blackening phenotype during the pupal stage. Dai [10] found that the expression level of the *aspartate decarboxylase (BmADC)* gene is significantly downregulated, affecting the conversion of DA to N-β-alanyl-dopamine (NBAD) and causing excessive accumulation of DA, ultimately resulting in the *bp* mutation in silkworms. Additionally, the expression of the *BmADC* gene is temperature-sensitive due to the influence of 11 heat shock factor binding sites within its regulatory region; at higher temperatures, expression levels are higher, resulting in a relatively lighter pupal color.

During the breeding of QiufengN, a variety resistant to the BmNPV virus, a mutant with deep black pupae was observed, named QiufengNBP. Some of the mutant pupae had difficulty shedding their pupal cuticle during metamorphosis, while some eggs developed normally, but the larvae could not hatch. These phenomena have not been observed in other black pupa mutants. Preliminary map-based cloning and sequence analysis by our research team indicated that the *ebony* gene may significantly influence the formation of the black pupa mutant and the expression of 30 KPs. The 30 K proteins (30 KPs) of silkworms belong to the lipoprotein 11 family and are defined as low-molecular-weight lipoproteins. These proteins have highly homologous amino acid sequences and are approximately 30 kDa in size, hence the name 30 KPs [11,12]. The synthesis of *B. mori* 30 KPs is regulated by juvenile hormone [12,13,14]. In silkworm oocytes, 30 KPs account for 35% of the yolk proteins but are used only during embryonic development. They are expressed in the fat body, larvae, and pupal epidermis [15,16]. The 30 KPs function as reservoirs of amino acids for de novo protein synthesis during embryonic development [17,18]. As storage proteins, 30 KPs also participate in energy transport and metabolic processes, such as the release of diacylglycerols and the transport of steroids and hormones [19]. Furthermore, 30 KPs can couple with glucose, dextran, maltose, and glycoproteins to form a defense mechanism [20,21]. To confirm the function of the *ebony* gene and its effect on 30 K protein expression during the pupal stage, this study used CRISPR/Cas9 gene-editing technology to knock out the *ebony* gene in the Nistari strain, confirming that the *ebony* gene significantly affects the formation of black pupae and the expression of 30 KPs.

## 2. Materials and Methods

### 2.1. Materials

The non-diapause silkworm strain Nistari and the non-diapause silkworm strain Nistari-Cas9, which has been introduced with the green fluorescent protein (*GFP*) and *cas9* gene, are both preserved by the Sericultural Research Institute, Jiangsu University of Science and Technology. The recombinant template plasmid PXL-IE1-dsred-U6-SgRNA1-U6-SgRNA2, the original plasmid PXL-IE1-dsred-U6-U6, and the helper plasmids Helper 08 and Helper 18 were all provided by the Institute of Plant Physiology and Ecology, Shanghai Institutes for Biological Sciences, Chinese Academy of Sciences. The template plasmid pCMV-T7 used for the synthesis of transposase gene mRNA was constructed by our laboratory.

### 2.2. CRISPR/Cas9

#### 2.2.1. Design of Knockout Sites

The CDS sequence of the target gene was downloaded from the silkworm website (http://sgid.popgenetics.net/index/searchlist, accessed on 1 May 2023). The SgRNA sites were designed using the CRISPRdirect online tool (http://crispr.dbcls.jp/, accessed on 1 May 2023) according to the sequence motifs 5′-GN19NGG-3′ or 5′-CCN19NC-3′. To ensure knockout efficiency, two SgRNA sites were designed. Primers were also designed on both sides of the SgRNA sites. Using the Nistari genome as a template, PCR amplification and sequencing were performed to confirm whether the sequence of the amplified product at the SgRNA sites matched the reference genome sequence. Two sites, site1 and site2 (Table S1), with matching sequences were selected for subsequent experiments.

#### 2.2.2. Construction of PXL-IE1-dsred-U6-SgRNA1-U6-SgRNA2 Recombinant Plasmid

Based on the SgRNA sites, site1 and site2, PCR primers Sg1F, Sg1R, Sg2F, and SgR (Table S1) were designed. PCR amplification was performed using an existing recombinant plasmid as a template to amplify SgRNA1 and SgRNA2 products, which were then purified using the MinElute Gel Extraction Kit 50 (QIAGEN, Hilden, Germany) according to the manufacturer’s instructions.

The PXL-IE1-dsred-U6-U6 plasmid was subjected to double digestion with Quickcut SalI (Takara, San Jose, CA, USA) and Quickcut NheI (Takara) restriction enzymes, and the linearized plasmid was recovered using the QIAquick Gel Extraction Kit 50 (QIAGEN).

Using the 2×MultiF Seamless Assembly Mix, the SgRNA1 and SgRNA2 fragments were ligated with the double-digested linearized plasmid PXL-IE1-dsred-U6-U6 to construct a new recombinant plasmid, PXL-IE1-dsred-U6-SgRNA1-U6-SgRNA2, which was then transformed into *Escherichia coli* DH5α competent cells. PCR was conducted on the transformed bacterial liquid using F3421 and R20 (Table S1) as upstream and downstream primers for verification. Following agarose gel electrophoresis, sequencing was performed with primer R3667 (Table S1). Plasmids with correct sequencing results were prepared in large quantities using the QIAGEN Plasmid Midi Kit 25 (QIAGEN) and stored at −20 °C for future use. The sequences of the primers used are shown in Table S1. Primer synthesis and DNA sequencing were completed by Sangon Biotech (Shanghai) Co., Ltd (Shanghai, China).

#### 2.2.3. Synthesis of Transposase mRNA

The pCMV-T7-Transposase plasmid was linearized by overnight digestion with SalI (Takara) and purified. Transposase mRNA was synthesized using the kit mMESSAGE mMACHINETM T7 Ultra (Thermo Fisher Scientific, Waltham, MA, USA) according to the manufacturer’s instructions, and the purified transposase mRNA was stored at −80 °C.

#### 2.2.4. Microinjection

The Nistari silkworm strain was reared until moth emergence. After mating for over 2 h in a bright environment, copulating moths were separated and allowed to lay eggs in a dark environment. Eggs that were 2–6 h old were brushed off from previous egg card coated with glue, washed with sterilized water to remove surface impurities, and then placed on a slide disinfected with 75% ethanol. A mixture of the recombinant plasmid PXL-IE1-dsred-U6-SgRNA1-U6-SgRNA2 in Section 2.2.2, helper plasmids Helper 08 and Helper 18, and the synthesized transposase mRNA in Section 2.2.3 was prepared at final concentrations of 400 ng/µL, 300 ng/µL, 300 ng/µL, and 200 ng/µL, respectively. This mixture was injected into the *B. mori* eggs using the TransferMan 4r micro-manipulation system (Eppendorf, Germany). After injection, the eggs were sealed with glue and incubated at 25 °C and 75–85% humidity until hatching, followed by rearing at 25–30 °C and 60–70% humidity.

### 2.3. Screening and Purification of Successfully Knocked Out ebony Gene Individuals

The injected Nistari silkworm eggs represented the G0 generation. After rearing to moth emergence, the G1 generation eggs were obtained by self-crossing. The G1 larvae that hatched from these eggs were screened under a fluorescence microscope for recombinant individuals showing discosoma sp. red fluorescent protein (*DsRed*). The G1 recombinant individuals were crossed with Niatari-Cas9 strain individuals to produce the G2 generation, which was then screened under a fluorescence microscope for dual fluorescence, exhibiting both *DsRed* and *GFP*. The dual-fluorescent individuals were reared until pupation, and the changes in pupal body color were observed. Upstream of site1, the forward primer ebF was designed, and downstream of site2, the reverse primer ebR was designed (Table S1). Using the genomic DNA of blackened G2 parents as the template, PCR amplification was performed. The PCR products were cloned and sequenced to determine the knockout form of the *ebony* gene in each G2 black pupa individual. Based on the sequencing results, G2 individuals that exhibited clear blackening were selected and crossed with wild-type Nistari to produce the G3 generation. The G3 population was screened for individuals that exhibited no fluorescence, which were then crossed with wild-type Nistari to produce the G4 generation. The G4 generation was reared in individual moth circles and self-crossed to produce the G5 generation. For the G5 generation, black pupa individuals from individual moth circles were selected for self-crossing to obtain a G6 generation of black pupae (NisBP) with only one knockout form, which could be stably inherited.

### 2.4. Construction of Expression Profile of ebony Gene After Gene Knockout

Material Preparation: Eggs of the G6 generation black pupa NisBP were incubated at 25 °C with 70–80% humidity. After hatching, the larvae were reared on fresh mulberry leaves at room temperature until pupation. During pupation, they were kept at 60–70% humidity and observed at 25 °C and 30 °C for blackening of the epidermis. At 25 °C, the pupal cuticle was collected at 2 h (beginning of blackening), 6 h, and 10 h (complete blackening) after pupation. For each time point, six replicates were taken, with three pupae in each replicate. Three of these replicates were used for two-dimensional protein electrophoresis experiments, and the other three were used for quantitative fluorescence experiments. Nistari was reared and sampled under the same conditions, and the samples were stored at −80 °C.

The pupal cuticle material was ground into powder in liquid nitrogen, and 1 mL of RNAiso Plus (TaKaRa) was added. Total RNA was extracted according to the manufacturer’s instructions. The concentration of RNA was measured using a Multiskan FC microplate reader (Thermo Fisher Scientific, America), and the quality of the RNA was assessed via electrophoresis. A total of 500 ng of RNA was used to synthesize cDNA through reverse transcription with the PrimeScript™ RT reagent Kit with gDNA Eraser (Takara), and the resulting cDNA was diluted to 100 ng/µL and stored at −20 °C. Based on the CDS sequence of the *ebony* gene, fluorescence quantitative primers were designed using Primer 6 software (Table S2). According to the BIO-RAD qRT-PCR kit instructions, the reaction system was prepared with the following components: iTaq Universal SYBR Green Supermix (2) 10 µL, forward and reverse primers 1 µL each, cDNA 1 µL, and RNase-free H_2_O 7 µL. The housekeeping gene used for normalization was *Actin 3* (GenBank ID: NM_001126254). The amplification program consisted of 95 °C for 1 min, followed by 40 cycles of 95 °C for 30 s and 60 °C for 30 s. The data obtained were analyzed using the 2^−∆∆Ct^ method [22] for relative quantification.

### 2.5. Two-Dimensional Analysis of Total Pupa Cuticle Proteins

Following the method described in Section 2.4, the pupal cuticles of G6 generation black pupa individuals (NisBP) and wild-type individuals (Nistari) were dissected at different developmental time points. The pupal cuticle material was rapidly ground into powder in liquid nitrogen, and 400 µL of sample lysis buffer (8 mol/L Urea, 2% CHAPS, Destreak, Shanghai, China) was added. The samples were placed on ice for 1 h to allow complete lysis, followed by centrifugation at 12,000 rpm for 10 min. The supernatant was collected and centrifuged again at 12,000 rpm for 10 min, and the resulting supernatant was used as the protein sample. The Bradford Protein Assay Kit was used to measure the protein concentration according to the manufacturer’s instructions. Protein standards (0.2 mg/mL) were added to a 96-well plate in volumes of 0, 2, 4, 6, 8, 12, 16, and 20 µL, and sample lysis buffer was added to bring the final volume to 20 µL. The test samples were added in volumes of 0.5, 1, 2, and 4 µL, and sample lysis buffer was added to bring the final volume to 20 µL. A total of 200 µL of 1 × G250 dye solution was added to each well, and the plate was incubated at room temperature for 3–5 min. Absorbance at 595 nm (A595) was measured using the Multiskan FC microplate reader (Thermo Fisher Scientific, America) to create a standard curve. The protein concentration of the test samples was calculated, and the samples were stored at −20 °C for later use.

For 2-DE, 200 µg of protein sample was mixed with 600 µL of hydration solution (8 mol/L Urea, 2% CHAPS, 1.2% Destreak, 0.5% IPG Buffer) until fully mixed and then added to an isoelectric focusing tank. Dry IPG strips (24 cm, pH 3–10) were placed with the gel side facing down, and mineral oil was added. First-dimension isoelectric focusing (1D-IEF) was performed by using a BIO-RAD PROTEN i12 IEF Cell isoelectric focusing instrument with the following program: 50 V 14 h, 250 V 0.5 h, 2000 V 1 h, 10,000 V 2 h, 10,000 V 6 h. After 1D-IEF, the IPG strip was equilibrated for 15 min in equilibrium buffer 1 (1.5 mol/L pH 8.8 Tris-Cl, 6 mol/L Urea, 30% Glycerol, 2% SDS, 0.002% Bromophenol Blue, 1% DTT), washed with ddH_2_O, and then equilibrated for 15 min in equilibrium buffer 2 (1.5 mol/L pH 8.8 Tris-Cl, 6 mol/L Urea, 30% Glycerol, 2% SDS, 0.002% Bromophenol Blue, 2% Iodoacetamide). After washing with ddH_2_O, the strip was placed on a 12% polyacrylamide separation gel for the second dimension of SDS-PAGE electrophoresis. After electrophoresis, the gel was stained using the silver staining method. The differential protein spots were excised and sent to Sangon Biotech (Shanghai) Co., Ltd. for mass spectrometry analysis.

### 2.6. Constructing the Expression Profile of Genes Corresponding to Differential Proteins

Based on the mass spectrometry identification results of the differential proteins, the coding sequences (CDS) of the corresponding differential genes were obtained. Fluorescent quantitative primers were designed (Table S2). Using the experimental materials and the methods from Section 2.4, the expression profiles of the genes corresponding to the differentially expressed proteins were analyzed.

## 3. Results

### 3.1. Construction of PXL-IE1-dsred-U6-SgRNA1-U6-SgRNA2 Transgenic Vector

**Figure 1:**
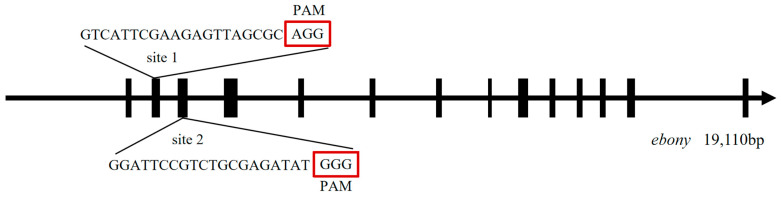
Structure of the *ebony* gene and SgRNA sites. The red box contains the Protospacer Adjacent Motif (PAM) sequence, which is responsible for recognizing and cleaving the target gene sequence, thereby enabling precise gene knockout.

The full length of the *ebony* gene is 19,110 bp and consists of 14 exons and 15 introns. Based on the characteristics of the *ebony* gene sequence, one SgRNA site (site1, site2) was designed on the second and third exons, respectively (Figure 1), using the online software CRISPRdirect. The PCR products of both sites were recombined with the linearized plasmid PXL-IE1-dsred-U6-U6 to construct the recombinant plasmid PXL-IE1-dsred-U6-SgRNA1-U6-SgRNA2 (Figure S1).

### 3.2. Acquisition of ebony Transgenic Silkworms

**Figure 2:**
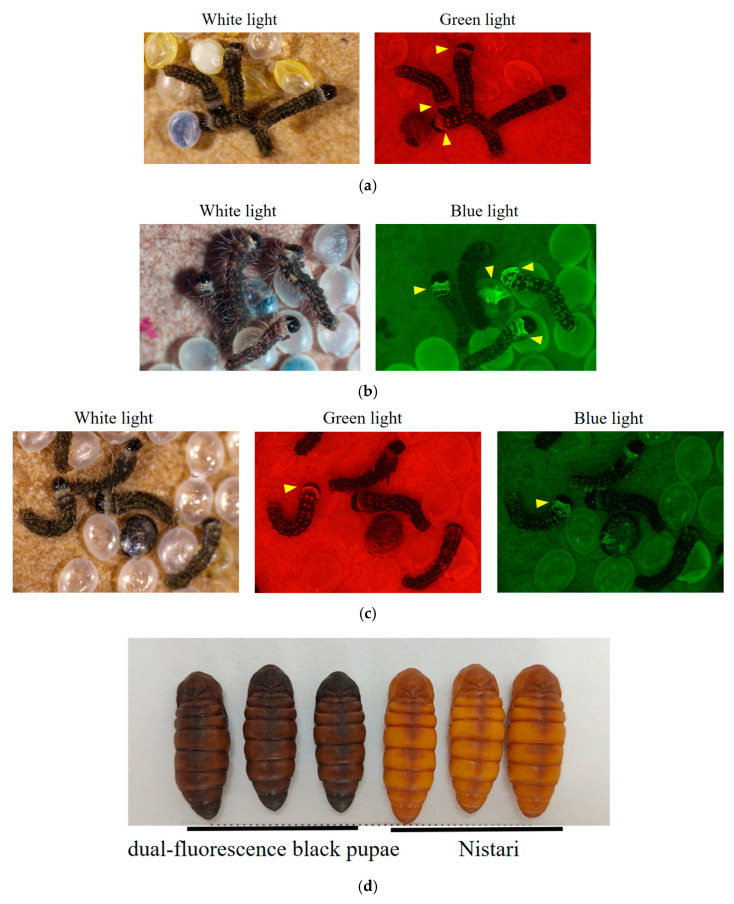
Phenotypes of blackened individuals in the G2 dual-fluorescent generation. (**a**) Positive G1 individual emitting *DsRed* (indicated by the arrow); (**b**) Nistari-Cas9 transgenic individual emitting *GFP* (indicated by the arrow); (**c**) G2 dual-fluorescent individual emitting both *DsRed* and *GFP* (indicated by the arrow); (**d**) a comparison of the phenotype of the dual-fluorescence black pupae and their wild-type Nistari counterparts at 36 h post-pupariation (left: dual-fluorescence black pupae; right: wild-type Nistari).

After injection, the G0 generation silkworms were self-crossed. The resulting G1 generation that just hatched silkworms was screened for individuals exhibiting *DsRed* under a fluorescence microscope (Figure 2a). The *DsRed* individuals were crossed with Nistari-Cas9 transgenic individuals (Figure 2b), and the G2 generation was screened for individuals displaying both *DsRed* and *GFP* (Figure 2c). The individuals who exhibited black pupae after pupation were identified as successful knockouts (Figure 2d).

### 3.3. Analysis of ebony Gene Knockout Forms

**Figure 3:**
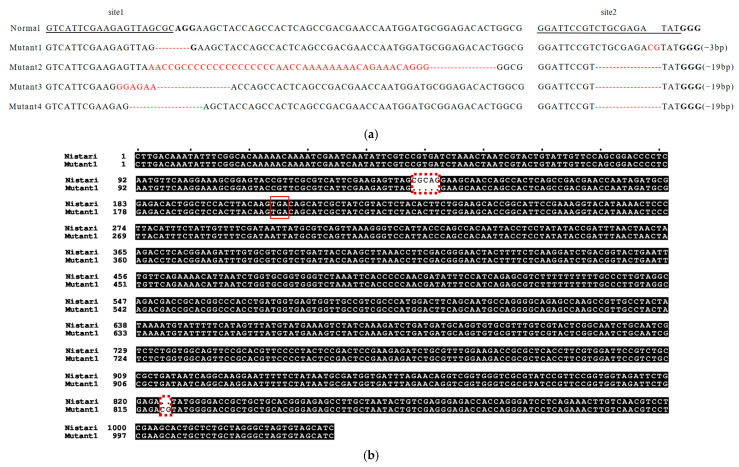
CRISPR/Cas9-mediated knockout forms of the *ebony* gene. (**a**) Different knockout forms mediated by CRISPR/Cas9 for the *ebony* gene; (**b**) post-knockout sequence structure of Mutant 1. The black underlines represent the sgRNA target sites, site1 and site2. The red bases indicate the mutated or inserted nucleotide sequences, and the red dashed lines represent the deleted nucleotide sequences. The dashed box highlights the sequence variation, while the red box shows the location of the stop codon after knockout.

To analyze the knockout forms of the *ebony* gene, the reference genome sequence of the *ebony* gene was used as a control. Ten double-fluorescent individuals were cloned and sequenced at the SgRNA sites. The results showed that four different length sequence variations occurred at the two SgRNA sites in the *ebony* gene of the 10 double-fluorescent individuals (Figure 3a). Among them, the sequencing results of Mutant 1 indicated that a 5 bp sequence (CGCAG) was deleted at the front of the second exon of the *ebony* cDNA sequence, causing a premature termination codon (TGA) to appear in the functional region of *ebony*, resulting in a nonsense mutation (Figure 3b). Therefore, Mutant 1 was further purified for subsequent research.

### 3.4. Purification of ebony Transgenic Silkworms

**Figure 4:**
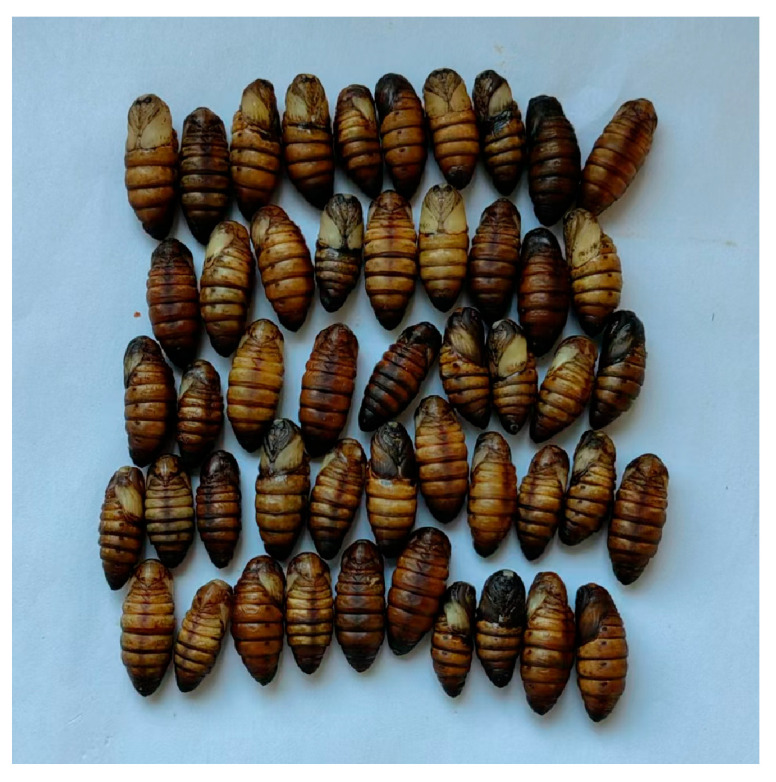
The phenotype of the G6 generation individuals (NisBP) at 36 h post-pupariation.

Mutant 1 was selected and crossed with the wild-type Nistari to continue rearing the G3 generation. G3 individuals that did not exhibit any fluorescence were crossed with wild-type Nistari to produce the G4 generation. Since all individuals in the G3 generation had normal pupal coloration, genomic DNA from the mating G3 individuals was extracted to ensure the selected G3 individuals contained the Mutant 1 knockout form. The G4 generation individuals were self-crossed within the moth circle to produce the G5 generation. In the G5 generation, single-batch rearing and self-crossing of the black pupae individuals within the same moth circle are used to produce G6 generation individuals, all of which were black pupae (NisBP) (Figure 4).

### 3.5. Analysis of the Blackening Process in NisBP and Comparison with Qiufeng NBP

**Figure 5:**
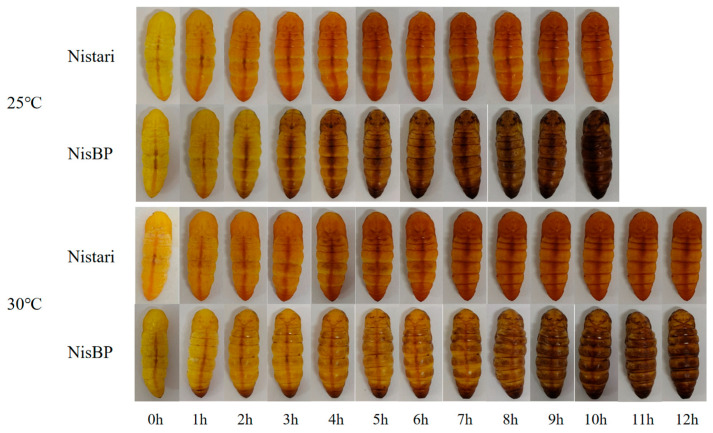
Pupae color changes in Nistari and NisBP at different temperatures and time points.

The blackening speed, coloring method, and color depth of NisBP varied under different temperatures (Figure 5). The results showed that NisBP was fully blackened at 25 °C after 10 h, with color starting from the vertical midline of the back, becoming fully colored after 2 h of pupation at the midline, and gradually coloring from the sides afterward. The midline continued to be the deepest colored, resulting in a blackish-brown color after the full blackening of the pupal body. At 30 °C, NisBP was fully blackened after 12 h, with less noticeable coloring along the vertical midline; the pupal body began to uniformly color after 3 h of pupation until fully colored, resulting in a brown color after the pupal body complete blackening.

The depth of coloring in the *ebony* knockout individual NisBP differed from that of the original black pupa mutant QiufengNBP. NisBP tended to be blackish-brown or brown after full blackening, while QiufengNBP was deep black after full blackening [23], which may be related to the strain and mutation form.

### 3.6. Tissue Expression Profile of the ebony Gene

**Figure 6:**
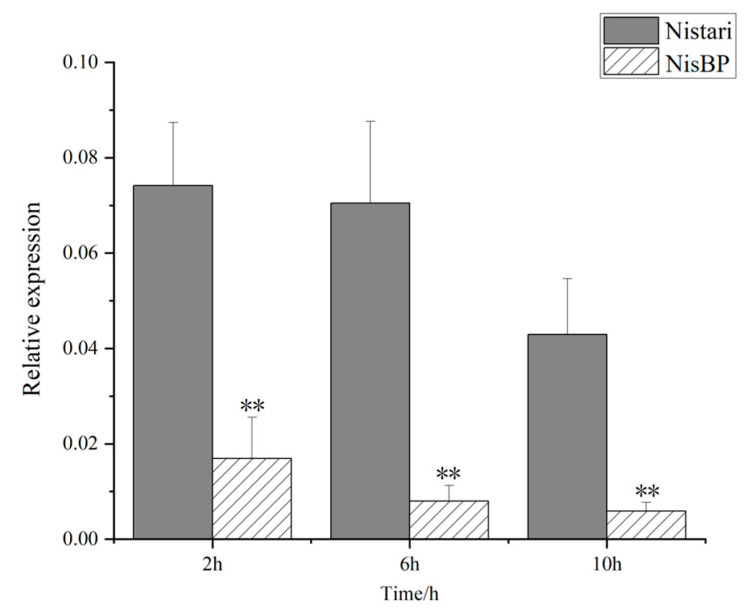
Expression of *ebony* gene in the pupal cuticle of Nistari and NisBP. The *t*-test was used for significance analysis (**: *p* < 0.01).

To investigate the expression of the *ebony* gene in NisBP, according to the method of 2.4, the tissue expression profiles of the *ebony* gene at different developmental time points were constructed for the G6 generation black pupa NisBP and its wild-type Nistari. The results showed that at 2 h, 6 h, and 10 h post-pupation, the expression levels of the *ebony* gene in the NisBP pupal cuticle were significantly lower than those in Nistari, and the expression level of the *ebony* gene gradually decreased as NisBP blackened (Figure 6).

### 3.7. Effects of ebony Gene Mutation on 30 K Protein Expression

**Figure 7:**
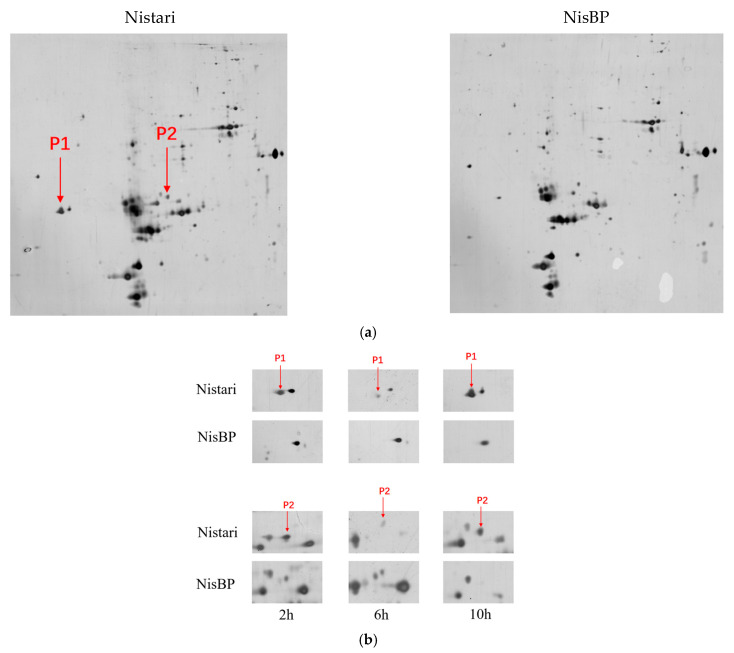
Two-dimensional analysis of total pupal proteins in Nistari and NisBP. (**a**) Expression of pupal proteins in Nistari and NisBP at 10 h; (**b**) expression of P1 and P2 in the pupal cuticle of Nistari and NisBP. P1 is highly expressed in Nistari at 2 h, 6 h, and 10 h after pupation but is undetectable in NisBP at all time points. P2 shows no significant difference in expression between Nistari and NisBP at 2 h and 6 h, but at 10 h, P2 is highly expressed in Nistari, while it is undetectable in NisBP.

To determine whether the *ebony* gene mutation affects the expression of pupal cuticle proteins, a 2-DE analysis of total protein expression in the pupal cuticle of G6 generation black pupae NisBP and their wild-type Nistari at different developmental stages was performed (Figure 7). The results showed that the protein expression trends in the pupal cuticle of Nistari and NisBP were generally consistent at the same time points (Figure S2). Two significant differential proteins were identified, with differential protein P1 being highly expressed in Nistari at all time points (2 h, 6 h, 10 h) post-pupation, whereas it was undetectable in the pupal cuticle of NisBP at all time points. Differential protein P2 shows no significant difference in the expression between Nistari and NisBP at 2 h and 6 h; however, at 10 h, P2 is highly expressed in Nistari, while it is undetectable in NisBP. Mass spectrometry identified both differential proteins as low-molecular-mass 30 kDa lipoprotein 19G1-like, corresponding to the genes *KWMTBOMO11904* and *KWMTBOMO11901*.

**Figure 8:**
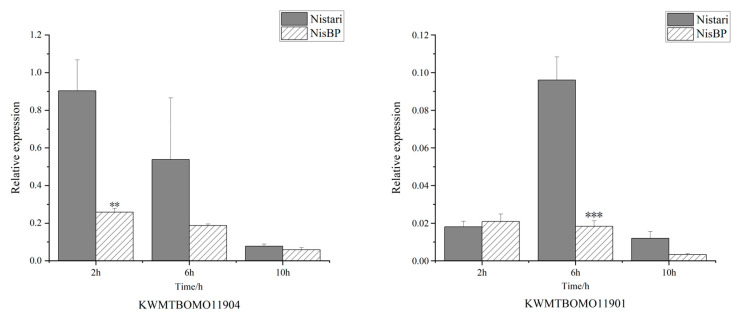
Expression levels of *KWMTBOMO11904* and *KWMTBOMO11901* genes in the pupal cuticle of Nistari and NisBP. The *t*-test was used for significance analysis (**: *p* < 0.01, ***: *p* < 0.001).

The expressions of *KWMTBOMO11904* and *KWMTBOMO11901* genes in the pupal cuticle of Nistari and NisBP at 2 h, 6 h, and 10 h after pupation were analyzed (Figure 8). The results showed that over time, the expression level of the *KWMTBOMO11904* gene gradually decreased in both Nistari and NisBP pupal cuticle, but at the same time points, the expression level of *KWMTBOMO11904* was consistently higher in Nistari compared to NisBP. The expression level of the *KWMTBOMO11901* gene in Nistari exhibited an initial increase followed by a decrease, while it remained low in the pupal cuticle of NisBP. The results indicated that gene expression was consistent with protein expression, confirming that the *ebony* mutation significantly impacted the expression of 30 KPs and their related genes during the pupal stage.

## 4. Discussion

There have been many studies on the *ebony* gene in insects. For example, in the lepidopteran insect, *Spodoptera litura*, the *ebony* gene is highly expressed during the pupal stage and the expression pattern was conserved, and CRISPR/Cas9 gene-editing technology successfully induced mutations in the *ebony* gene, leading to the formation of black pupae [24]. The *ebony* gene is expressed at various developmental stages in the lepidopteran insect, *Chilo suppressalis*, with a high expression during the pupal and head stages. After knocking out the *ebony* gene using CRISPR/Cas9 technology, the *Csebony* mutant exhibited deeper coloration than the wild-type throughout its development, especially during the pupal stage, confirming that the knockout of the *ebony* gene directly led to pupal melanization [25]. In the silkworm, the *so* mutation leads to a smoke-colored larval body and black pupae. The gene controlling the blackening of the *so* mutant is located at the end of genetic linkage group 26 in the silkworm. Studies by Futahashi found that the *ebony* gene is closely linked to the *so* locus, with large deletions found in the C-terminal region of the *ebony* gene ORF in the *so* mutants, altering the function of the *ebony* gene and resulting in the blackening phenotype throughout the silkworm’s development, especially during the pupal stage [6,26]. Our previous research on the black pupa mutant QiufengNBP indicated that the formation of the QiufengNBP black pupa mutant might be due to a mutation in the *ebony* gene [23]. Therefore, this experiment performed a knockout of the *ebony* gene, confirming that it plays a key role in black pupa formation. This experiment found that at different temperatures, the speed of melanization, coloration pattern, and color intensity of *ebony* gene knockout individuals vary. However, there are currently no studies on how temperature affects the expression of the *ebony* gene, which remains to be explored in the future. Since the molecular mechanisms underlying the entire black pupa formation are relatively complex, the expression levels of pupal cuticle proteins were analyzed using 2-DE. The results indicate that the *ebony* gene significantly influences the expression of 30 KPs in the pupal cuticle.

The 30 K proteins in silkworms were first isolated from the hemolymph of the 5th instar to pupation by Izumi [17]. They are mainly synthesized in the fat body of 5th instar silkworms and subsequently secreted into the hemolymph, where they exist in large amounts. During the growth and development of silkworms, 30 KPs play significant roles. After 3 days post-5th instar, 30 KPs are strongly expressed [27]. During pupation, 30 KPs accumulate substantially in the hemolymph and are then gradually transferred to the ovaries, absorbed by oocytes, and stored in silkworm eggs [28]. During embryonic development, the 30 KPs in *B. mori* eggs are gradually degraded by proteases, providing nutrients for embryonic development. They serve as storage proteins, capable of storing amino acids and utilizing them for protein synthesis during embryonic development [29,30]. Our previous research on the black pupa mutant QiufengNBP and its wild-type QiufengN conducted 2-DE and transcriptome analyses, revealing 12 differentially expressed genes in the 30 KPs family, including *KWMTBOMO11904* and *KWMTBOMO11901*. This suggests that the emergence of the black pupa mutant QiufengNBP might affect the expression of silkworm 30 KPs, leading to the hypothesis that the *ebony* gene may influence the expression of 30 KPs during the pupal stage [23]. This study confirms that the *ebony* gene in silkworms significantly affects the expression of 30 KPs during the pupal stage, which may directly or indirectly impact the growth and development processes of silkworms, resulting in the blackening of the pupal body. These findings are consistent with our previous research results. Although studies have shown that *B. mori* 30 KPs possess functions such as inhibiting the apoptosis of insects and human cells [31], and antifungal properties [32], no research has yet been conducted on how 30 KPs participate in black pupa formation. Further research will be conducted in the future.

## 5. Conclusions

The results indicate that the *ebony* gene not only plays a key role in black pupa formation but also significantly influences the expression of 30 KPs closely related to the growth and development of silkworms. Therefore, studying the effects of the *ebony* gene on 30 KPs is of great significance for improving the functional understanding of 30 KPs and enriching the molecular mechanisms of growth and development, warranting further investigation.

## Data Availability

The data presented in this study are available in the Supplementary Materials files.

## Supplementary Materials

The following supporting information can be downloaded at https://www.mdpi.com/article/10.3390/genes15121560/s1. Table S1: Primer sequences used in PCR and nucleic acid sequences involved in experiments; Table S2: Primers used in qRT-PCR; Figure S1: Transgenic vector PXL-IE1-dsred-U6-SgRNA1-U6-SgRNA2; Figure S2: Two-dimensional electrophoresis of pupae cuticle of Nistari and NisBP at 2 h, 6 h, and 10 h.

## References

[1] Gorman M.J., An C., Kanost M.R. (2007). Characterization of tyrosine hydroxylase from Manduca sexta. Insect Biochem. Molec..

[2] Li X.Y., Fan D.D., Zhang W., Liu G.C., Zhang L., Zhao L., Fang X.D., Chen L., Dong Y., Chen C. (2015). Outbred genome sequencing and CRISPR/Cas9 gene-editing in butterflies. Nat. Commun..

[3] Li M., Bui M., Yang T., Bowman C.S., White B.J., Ak-bari O.S. (2017). Germline Cas9 expression yields highly efficient genome engineering in a major worldwide disease vector, Aedes aegypti. Proc. Natl. Acad. Sci. USA.

[4] Rylee J.C., Siniard D.J., Doucette K.K., Zentner G.E., Zel-hof A.C. (2018). Expanding the genetic toolkit of Tribolium castaneum. PLoS ONE.

[5] Wittkopp P.J., True J.R., Carroll S.B. (2002). Reciprocal functions ofthe Drosophila Yellow and Ebony proteins in the development and evolution ofpigment patterns. Development.

[6] Futahashi R., Sato J., Meng Y., Okamoto S., Daimon T., Yamamoto K., Suetsugu Y., Narukawa J., Takahashi H., Banno Y. (2008). yellow and ebony are the responsible genes for the larval color mutants of the silkworm *Bombyx mori*. Genetics.

[7] Lu C., Dai F.Y., Xiang Z.H. (2003). Studies on the mutation strains of the *Bombyx mori* gene bank. Sci. Sin..

[8] Dai F.Y., Qiao L., Tong X.L., Cao C., Chen P., Chen J., Lu C., Xiang Z.H. (2010). Mutations of an arylalkylamine-N-acetyltransferase, Bm-iAANAT, are responsible for silkworm melanism mutant. J. Biol. Chem..

[9] Zhan S., Guo Q., Li M., Li M., Li J., Miao X., Huang Y. (2010). Disruption of an N-acetyltransferase gene in the silkworm reveals a novel role in pigmentation. Development.

[10] Dai F.Y., Qiao L., Cao C., Liu X.F., Tong X.L., He S.Z., Hu H., Zhang L., Wu S.Y., Tan D. (2015). Aspartate decarboxylase is required for a normal pupa pigmentation pattern in the silkworm, *Bombyx mori*. Sci. Rep..

[11] Hou Y., Zou Y., Wang F., Gong J., Zhong X., Xia Q., Zhao P. (2010). Comparative analysis of proteome maps of silkworm hemolymph during different developmental stages. Proteome Sci..

[12] Ogawa N., Kishimoto A., Asano T., Izumi S. (2005). The homeodomain protein PBX participates in JH-related suppressive regulation on the expression of major plasma protein genes in the silkworm, *Bombyx mori.*. Insect Biochem. Molec..

[13] Mori S., Izumi S., Tomino S. (1991). Structures and organization of major plasma protein genes of the silkworm *Bombyx mori*. J. Mol. Biol..

[14] Sakai N., Mori S., Izumi S., Haino-Fukushima K., Ogura T., Maekawa H., Tomino S. (1988). Structures and expression of mRNAs coding for major plasma proteins of *Bombyx mori*. BBA Gene Struct. Expr..

[15] Zhang Y., Dong Z., Liu S., Yang Q., Zhao P., Xia Q. (2012). Identification of novel members reveals the structural and functional divergence of lepidopteran-specific Lipoprotein_11 family. Funct. Integr. Genom..

[16] Zhu J., Indrasith L.S., Yamashita O. (1986). Characterization of vitellin, egg-specific protein and 30 kDa protein from Bombyx eggs, and their fates during oogenesis and embryogenesis. BBA Gen. Subj..

[17] Izumi S., Fujie J., Yamada S., Tomino S. (1981). Molecular properties and biosynthesis of major plasma proteins in *Bombyx mori*. BBA Protein Struct..

[18] Mine E., Izumi S., Katsuki M., Tomino S. (1983). Developmental and sex-dependent regulation of storage protein synthesis in the silkworm, *Bombyx mori*. Dev. Biol..

[19] Chapman M. (1980). Animal lipoproteins: Chemistry, structure, and compara-tive aspects. J. Lipid Res..

[20] Ujita M., Katsuno Y., Kawachi I., Ueno Y., Banno Y., Fujii H., Hara A. (2005). Glucan-binding activity of silkworm 30-kDa apolipoprotein and its involvement in defense against fungal infection. Biosci. Biotech. Bioch..

[21] Ueno Y., He N., Ujita M., Yamamoto K., Banno Y., Fujii H., Aso Y. (2006). Silkworm midgut proteins interacting with a hemolymph protease inhibitor, CI-8. Biosci. Biotech. Bioch..

[22] Livak K.J., Schmittgen T.D. (2001). Analysis of relative gene expression data using real-time quantitative PCR and the 2-∆∆Ct method. Methods.

[23] Sun J., Zheng X., Ouyang G., Qian H.Y., Chen A.L. (2023). Ebony plays an important role in egg hatching and 30k protein expression of silkworm (*Bombyx mori*). Arch. Insect Biochem..

[24] Bi H.L., Xu J., He L., Zhang Y., Li K., Huang Y.P. (2019). CRISPR/Cas9-mediated ebony knockout results in puparium melanism in Spodoptera litura. Insect Sci..

[25] Sun H., Huang J.M., Liu Y., Ge W.C., Wang S., Yang F.X., Gao C.F., Wu S.F. (2021). Knockout of ebony gene leads to melanin pigmentation in the rice stem borer, *Chilo suppressalis* (Lepidoptera: Crambidae). Acta Entomol. Sin..

[26] Banno Y., Kawaguchi Y., Shokyu I., Doira H. (1989). Linkage studies of *Bombyx mori*: Discovery of the twenty-sixth linkage group, sooty and non-molting of Ishiko. J. Seric. Sci. Jpn..

[27] Zhong B.X., Li J.K., Lin J.R., Liang J.S., Su S.K., Xu H.S., Yan H.Y., Zhang P.B., Fujii H. (2005). Possible effect of 30K proteins in embryonic development of silkworm *Bombyx mori*. Acta Bioch. Bioph. Sin..

[28] Chen Y.L., Yamashita O. (1990). Nonselective uptake of different 30kDa plasma proteins by developing ovaries of the silkworm, *Bombyx mori*. J. Seric. Sci. Jpn..

[29] Ji M.M., Liu A.Q., Gan L.P., Xing R., Wang H., Sima Y.H., Xu S.Q. (2013). Functional analysis of 30K proteins during silk gland degeneration by a caspase-dependent pathway in Bombyx. Insect Mol. Biol..

[30] Shi X.F., Li Y.N., Yi Y.Z., Xiao X.G., Zhang Z.F. (2015). Identification and characterization of 30 K protein genes found in *Bombyx mori* (Lepidoptera: Bombycidae) transcriptome. J. Insect Sci..

[31] Park H.J., Kim E.J., Koo T.Y., Park T.H. (2003). Purification of recombinant 30K protein produced in Escherichia coli and its anti-apoptotic effect in mammalian and insect cell systems. Enzym. Microb. Tech..

[32] Ye L., Zhang Y., Dong Z.M., Guo P.C., Zhao D.C., Li H.Y., Hu H., Zhou X.F., Chen H.Q., Zhao P. (2021). Five Silkworm 30K Proteins Are Involved in the Cellular Immunity against Fungi. Insects.

