# Supplementary material for: The ebony Gene in Silkworm Black Pupae Significantly Affects 30 K Proteins During the Pupal Stage
Source: Genes (Basel). 2024 Nov 29;15(12):1560. doi: 10.3390/genes15121560 (PMC11675696; doi:10.3390/genes15121560)
Supplement: Supplementary file 1 [file genes-15-01560-s001.zip › genes-3294588-supplementary.pdf]

Table S1. Primer sequences used in PCR and nucleic acid sequences involved in experiments.

Sequence name	Sequences	Purposes
site 1	GTCATTCGAAGAGTTAGCGC	SgRNA sites
site 2	GGATTCCGTCTGCGAGATAT	
Sg1F	AATATCGTGCTCTACAAGTGTCATTCGAAGAGTTAGCGCGTTTTAGAGCTAGAAATAGC	Used to amplify SgRNA1
Sg1R	ATATCTCGCAGACGGAATCCACTTGTAGAGCACGATATT	
Sg2F	GGATTCCGTCTGCGAGATATGTTTTAGAGCTAGAAATAGC	Used to amplify SgRNA2
SgR	TATAGATATCAAGCTGCTAGAAAAAAAAGCACCGACTCGG	
F3421	GTGGAGCTCCAGCTTTTGTT	Used to verify agarose gel electrophoresis bands
R20	AAAAAAAAGCACCGACTCGG	
R3667	GTGAGTCAAAATGACGCATG	Used for sequencing validation
ebF	CTTAGTTAGATTGACGGCGTTG	Used for sequencing validation
ebR	CCATCGGACCTATGAAAGTTCC	

Table S2. Primers used in qRT-PCR.

Genes	Primer sequences (5'→3')
Actin 3	F: GGATGTCCACGTCGCACTT<br>R: GCGCGGCTACTCGTTCACT
ebony	F: CACGGAACCCTCCACGTAC<br>R: GAACAGCGAACCAGTAGCAG
KWMTBOMO11901	F: TCTACGAGGACAAGAAGAGCGAAGT<br>R: CTTTGGAGCCTTGGAGCCATAGC
KWMTBOMO11904	F: TCGTCCGTGATTGTTTCCCAGTTG<br>R: CTGCCATCGTTGCCGTGAACA

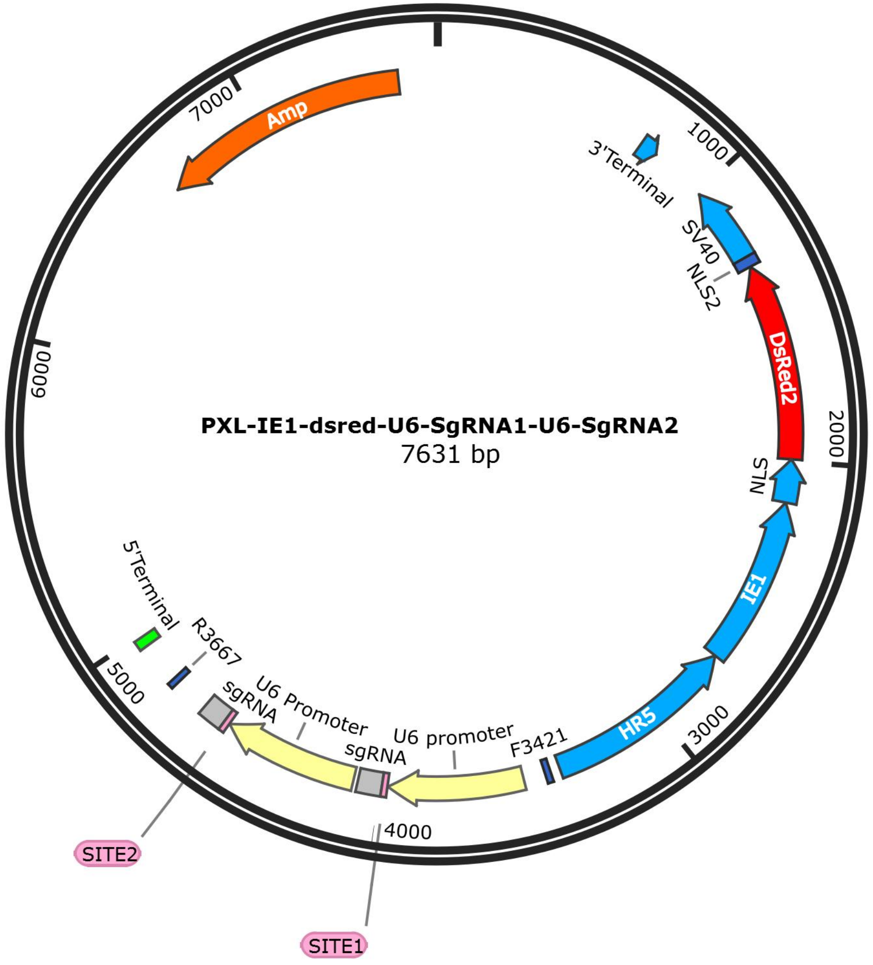

Figure S1. Transgenic vector PXL-IE1-dsred-U6-SgRNA1-U6-SgRNA2.

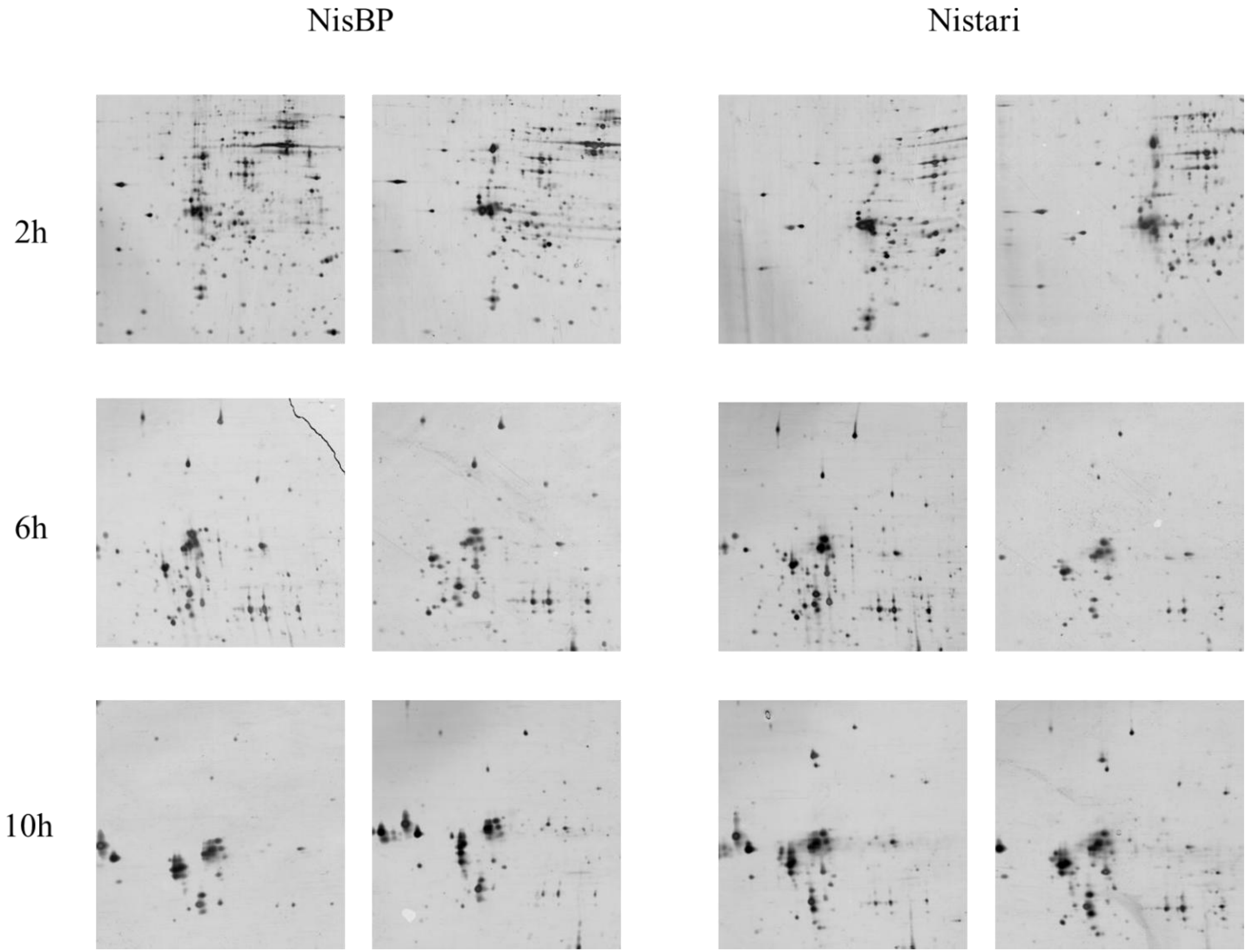

Figure S2. Two-dimensional electrophoresis of pupae cuticle of Nistari and NisBP at 2h, 6h, and 10h
